# Population‐Based Survival Analysis of Solitary Plasmacytoma of Spine in the United States From 2000 to 2020

**DOI:** 10.1002/cnr2.70299

**Published:** 2025-08-05

**Authors:** Kevin E. Agner, Luke G. Comisford, Alec G. Kotler, Jacob A. Wells, Michael C. Larkins

**Affiliations:** ^1^ The Ohio State University College of Medicine Columbus Ohio USA; ^2^ Department of Emergency Medicine Boonshoft School of Medicine at Wright State University Fairborn Ohio USA

**Keywords:** cancer epidemiology, radiation, SEER, solitary plasmacytoma, spinal plasmacytoma, surgical oncology

## Abstract

**Background:**

Solitary plasmacytomas of bone of the spine (SPBS) are rare tumors associated with significant morbidity and mortality, especially with progression to multiple myeloma.

**Aims:**

While demographic and treatment factors influencing survival have been investigated in previous studies, analysis using the most recent population data and assessing more granular data such as the extent of surgical resection in conjunction with radiotherapy has yet to be performed.

**Methods:**

The Surveillance, Epidemiology, and End Results (SEER) Program was queried for patients with a diagnosis of SPBS (ICD‐0‐3 code 9731/3). Demographic and treatment variables were analyzed using Cox regression, and a log‐rank analysis was used to assess 5‐year overall survival (5y OS).

**Results:**

A total of 1391 patients diagnosed with localized SPBS were identified. Multivariate analysis revealed that increasing age at diagnosis (HR = 2.25, *p* < 0.001) and higher income (HR = 1.471; *p* < 0.001) were significantly associated with decreased 5y OS, while being married was associated with improved survival (HR = 0.671, *p* < 0.001). Furthermore, treatment with radiation therapy (HR = 0.613, *p* < 0.001) and gross total resection (HR = 0.657; *p* = 0.020) were associated with improved survival outcomes. Univariate analysis confirmed the demographic factor significance, with radiotherapy combined with surgery being associated with improved survival versus sole radiotherapy (49.4% vs. 40.8%; *p* = 0.004).

**Conclusion:**

This population‐based analysis of SPBS highlights key prognostic factors for 5y OS. Increased age, higher income, and single marital status were associated with worse outcomes, while radiation therapy and greater extent of surgical procedure improved survival. Notably, radiotherapy combined with surgery showed better survival than radiation alone, challenging current national guidelines that recommend radiotherapy with or without surgery. Additionally, gross total resection demonstrated the highest overall survival among the various surgical procedures. These findings suggest that incorporating combined therapy into treatment protocols could improve outcomes and refine future guidelines.

## 1 Introduction

Plasmacytoma is a rare hematologic condition characterized by the monoclonal proliferation of plasma cells that form localized tumors. Solitary plasmacytoma of bone of the spine (SPBS) specifically refers to the formation of a single tumor at a distinct site within the vertebra [1]. Solitary plasmacytomas of bone account for about 5% of all plasma cell disorders, with approximately 70% of cases occurring in the spine, making SPBS one of the most common primary malignant bone tumors of the spine [2, 3, 4]. The median patient survival duration of SPBS is 7.5–12 years, and approximately 50% of SPBS patients develop multiple myeloma (MM) within 2–3 years of initial diagnosis [5].

SPBS has historically had a low detection rate; however, its incidence has gradually increased with improvements in diagnosis and understanding. The current National Comprehensive Cancer Network (NCCN) guidelines recommend radiotherapy ± surgery, depending on spinal stability and risk of progression [6]. Radiotherapy has provided a high response rate of up to 80% in clinical trials [7]. Surgery is less common but more specific for vertebral collapse, spinal instability, or spinal cord compression cases [5].

A review of the Surveillance, Epidemiology, and End Results (SEER) database, a population‐based program in the United States that collects survival data on cancer, demonstrated that surgery combined with radiotherapy reduces progression to MM in patients with SPBS compared to radiotherapy alone [2, 8]. Surgery alone was associated with worse survival outcomes compared to radiation‐based therapies, and survival was not analyzed among the different surgical approaches [5]. Additionally, studies using SEER have also demonstrated that there is a higher risk of SPBS and progression from SPBS to MM due to increased age [7].

The rising incidence and prevalence of SPBS make a robust understanding of implications surrounding SPBS treatment options even more urgent. There is considerable controversy surrounding the prognosis and risk factors for SPBS, with differing opinions in recent research [9, 10]. While several studies have evaluated the progression from SPBS to MM and offered critical risk information regarding SPBS treatment modalities, no studies to date have specifically explored the prognosis of SPBS based on the extent of surgical resection of the tumor. This study aims to fill this gap by conducting a retrospective epidemiologic survival study of SPBS. A major gap in the research is also observed in the lack of clarity regarding the role of radiotherapy with and without surgery in the treatment of SPBS, as the NCCN guidelines offer limited guidance on whether radiotherapy alone or in combination with surgery provides the best outcomes, leaving this decision largely at the discretion of clinicians [6]. Enhancing established research and utilizing the SEER database, this study will analyze the survival outcomes of various SPBS patients, across variable demographics, and evaluate the relationship between the type of tumor removal and prognosis. Considering the high risk of progression to MM and significant morbidity associated with SPBS, current treatment guidelines remain ambiguous regarding the optimal role of surgical resection and combined therapy, highlighting the critical need for this large‐scale, population‐based survival analysis to inform evidence‐based clinical decision‐making. The foregoing information could provide a valuable reference to clinicians and patients as they assess treatment options.

## 2 Materials and Methods

### 2.1 Patient Identification

Patients diagnosed with SPBS were identified through the SEER Program's 17‐registry incidence dataset, which includes United States, multicenter cases reported between 2000 and 2020 [8]. Cases were selected using the International Classification of Diseases for Oncology, Third Edition (ICD‐O‐3) code 9731/3, corresponding to “Solitary plasmacytoma of bone.” Inclusion criteria mandated availability of patient age data. Data was further filtered to include cases with a primary site designated as “vertebra” and a stage classified as “localized.”

### 2.2 Data Analysis

Statistical analyses were performed using SPSS software (version 29.0; IBM Corp., Armonk, NY). Statistical significance was defined as a *p* value of < 0.05, with 95% confidence intervals (CIs) presented in brackets [95% CI]. Multivariate analysis utilized Cox proportional hazards regression to evaluate 5‐year overall survival (5y OS). Covariates included in the final Cox regression model were: age, sex, race, marital status, income, chemotherapy, RT, and surgical procedure. Kaplan–Meier (KM) survival curves were constructed and compared using log‐rank tests for univariate and subgroup analyses. Unknown data for all variables were excluded from both multivariate and univariate analyses to maintain data integrity. While reported in frequency tables, the unknown data accounted for less than 5% of the dataset in all variables included in the multivariate analysis. Surgical classifications were assigned based on guidelines outlined in the 2021 SEER Staging Manual. Income data were derived from census‐tract level estimates, and income brackets were categorized based on the dataset median, with groups split into $40 000–$74 999 and $75 000–$120 000+. For regression analysis, marital status was dichotomized into “Married” and “Single,” with the latter category including divorced, separated, widowed, and never married patients.

## 3 Results

### 3.1 Cohort Overview

**FIGURE 1:**
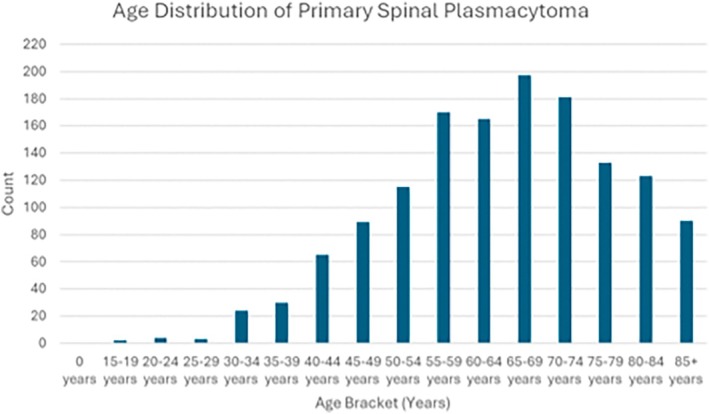
Distribution of solitary plasmacytoma of bone of the spine by age. Incidence of solitary plasmacytoma of bone of the spine by five‐year age bracket, as reported in data from the Surveillance, Epidemiology, and End Results (SEER) Program among patients in the United States, diagnosed between 2000 and 2020.

A total of 1391 patients with SPBS of localized stage were identified. The median age at diagnosis, based on five‐year age intervals, was 60–64 years (Figure 1). Age‐specific case distribution is illustrated in Figure 1. Detailed demographic and disease characteristics are summarized in Table 1. Patient age was divided into two groups to allow for strong statistical comparison (Table 2).

**TABLE 1:** Demographic and disease characteristic information for patients diagnosed with solitary plasmacytoma of bone of the spine in the United States between 2000 and 2020, as identified via the Surveillance, Epidemiology, and End Results (SEER) Program.

Variable	Number (% of cohort; *n* = 1391)
*Age at diagnosis (years)*	
0–64	667 (48.0)
65–85	724 (52.0)
*Sex*	
Female	513 (36.9)
Male	878 (63.1)
*Race*	
American Indian/Alaska Native	10 (0.7)
Asian	55 (4.0)
Black	211 (15.2)
White	1108 (79.7)
Unknown	7 (0.5)
*Marital status*	
Single	453 (32.6)
Married	880 (63.3)
Unknown	58 (4.2)
*Marital status subgroup*	
Married	880 (63.3)
Divorced	108 (7.8)
Separated	13 (0.9)
Single (never married)	192 (13.8)
Unknown	58 (4.2)
Widowed	140 (10.1)
*Median yearly income*	
$40 000–$74 999	667 (48.0)
$75 000–$120 000+	724 (52.0)

**TABLE 2:** Treatment characteristics of patients diagnosed with solitary plasmacytoma of bone of the spine in the United States between 2000 and 2020 as identified in the SEER database.

Variable	Number (% of cohort; *n* = 1391)
*Radiation*	
No radiation	232 (16.7%)
Radiation	1139 (81.9)
Unknown	20 (1.4)
*Chemotherapy*	
No chemotherapy/unknown	1175 (84.5)
Chemotherapy	216 (15.5)
*Surgical procedure*	
None	909 (65.3)
Tumor destruction/non‐resection	15 (1.1)
Subtotal resection	365 (26.2)
Gross total resection	78 (5.6)
Surgery NOS	24 (1.7)
*Combined treatment*	
Radiotherapy without surgery	750 (53.9)
Radiotherapy with surgery	389 (28.0)
Unknown	252 (18.1)
*Radiation alone vs. surgery alone*	
Radiation alone	750 (53.9)
Surgery alone	86 (6.2)

### 3.2 Demographic Factor Analysis

Multivariate Cox regression analysis demonstrated that increasing age at diagnosis was significantly associated with decreased 5y OS (hazard ratio [HR] = 2.25; *p* < 0.001; Table 3). This finding was further supported by univariate analysis, which showed a significant difference in 5y OS rates between age groups, with patients aged 0–64 years exhibiting a better 5y OS (54.3% [50.49–58.05]) compared to those aged 65–85+ (27.5% [24.23–30.74], *p* < 0.001; Table 4). Multivariate Cox regression analysis showed no significant association between sex and 5y OS (HR = 1.024; *p* = 0.755) (Table 3). Univariate analysis showed a slight trend in survival differences between females (37.2% [33.05–41.42]) and males (42.1% [38.87–45.41]) which resulted in a statistically significant *p* value (*p* = 0.042) (Table 4). Race was not shown to be statistically significant in either analysis. The multivariate analysis showed no significant differences for Asian (HR = 1.389, *p* = 0.496), Black (HR = 1.416, *p* = 0.447) and White patients (HR = 1.262; *p* = 0.605) relative to all race groups (Table 3). In univariate analyses, White (41.2% [38.35–44.14]), Black (37.0% [30.45–43.48]), American Indian/Alaska Native (50.0% [19.01–80.99]), and Asian American (36.4% [23.65–49.08]) survival rates resulted in a nonsignificant *p* value (*p* = 0.747; Table 4). Multivariate Cox regression analysis found that married patients had significantly improved 5y OS when compared to single patients (HR = 0.671; *p* < 0.001) (Table 3). Univariate analysis reaffirmed these findings, with married patients exhibiting better 5y OS (44.5% [41.26–47.83]) compared to single patients (32.2% [27.93–36.53], *p* < 0.001). Subgroup analysis demonstrated that married patients did have improved 5y OS when compared to divorced (29.6% [21.02–38.24]) separated (53.8% [26.75–80.95]) single (never married) patients (39.1% [32.16–45.96]), and widowed patients (22.9% [15.90–29.81], *p* < 0.001; Table 4). Multivariate Cox regression analysis demonstrated that patients with lower income had an improved 5y OS (HR = 1.471; *p* < 0.001) (Table 3). Univariate analysis also demonstrated a significantly improved 5y OS in patients with incomes between 40 000–74 999 (46.3% [42.54–50.11]) when compared to incomes at 75 000–120 000+ (34.8% [31.34–38.28], *p* < 0.001) (Table 4).

**TABLE 3:** Cox regression analysis of 5‐year overall survival among patients with solitary plasmacytoma of bone of the spine.

Variables	*p*	Hazard ratio
Age (65–85+ vs. 0–64 years at diagnosis)^a^	< 0.001	2.25
Sex (female vs. male)	0.76	1.02
*Race*	0.62	—
Asian or Pacific Islander	0.50	1.39
Black	0.45	1.42
White	0.61	1.26
Income ($75 000–$120 000+ vs. $40 000–$74 999)	< 0.001	1.47
Marital status (married vs. single)	< 0.001	0.67
Radiation (yes vs. no)	< 0.001	0.61
Chemotherapy (yes vs. no)	0.34	0.91
*Surgical procedure*	0.013	—
Tumor destruction/non‐resection	0.40	0.71
STR	0.16	0.89
GTR	0.02	0.66
Surgery NOS	0.03	1.73

*Note:* Significant variables include age, income, marital status, radiation, and surgical procedures. Reference categories were as follows: age 0–64 years, male sex, American Indian/Alaska Native race, income $40 000–$74 999, single status, no radiation, no chemotherapy, and no surgery for surgical procedure comparisons.

Abbreviation: NOS, not otherwise specified.

^a^ Age at diagnosis with solitary plasmacytoma of bone of the spine.

**TABLE 4:** Kaplan–Meier univariate analysis of 5‐year overall survival among patients with solitary plasmacytoma of bone of the spine.

Variables	OS [95% CI]	Log‐rank *p* value
*Age at diagnosis (years)*		< 0.001
0–64	54.30% [50.49–58.05]	
65–85+	27.50% [24.23–30.74]	
*Sex*		0.042
Female	37.20% [33.05–41.42]	
Male	42.10% [38.87–45.41]	
*Race*		0.75
White	41.20% [38.35–44.14]	
Black	37.00% [30.45–43.48]	
American Indian/Alaska Native	50.00% [19.01–80.99]	
Asian American	36.40% [23.65–49.08]	
*Marital status*		< 0.001
Single	32.20% [27.93–36.53]	
Married	44.50% [41.26–47.83]	
*Marital status subgroups*		< 0.001
Divorced	29.60% [21.02–38.24]	
Married	44.50% [41.26–47.83]	
Separated	53.80% [26.75–80.95]	
Single, never married	39.10% [32.16–45.96]	
Widowed	22.90% [15.90–29.81]	
*Income*		< 0.001
< $40 000–$74 999	46.30% [42.54–50.11]	
$75 000–$120 000+	34.80% [31.34–38.28]	
*Chemotherapy*		0.46
No chemotherapy	40.10% [37.28–42.89]	
Chemotherapy	41.70% [35.09–48.24]	
*Surgical procedure*		0.002
No surgery	37.50% [34.37–40.66]	
Tumor destruction/non‐resection	53.30% [28.09–78.58]	
Subtotal resection	44.40% [39.29–49.48]	
Gross total resection	56.40% [45.41–67.42]	
Surgery NOS	25.00% [7.68–42.32]	
*Radiation*		< 0.001
No radiation	25.00% [19.43–30.57]	
Radiation	43.70% [40.84–46.60]	
*Radiation* ± *surgery*		0.004
Radiation without surgery	40.80% [37.28–44.32]	
Radiation with surgery	49.40% [44.39–54.33]	
*Radiation alone vs. surgery alone*		0.28
Radiation alone	59.20% [55.68–62.72]	
Surgery alone	69.80% [60.06–79.47]	

*Note:* Age, sex, marital status, income, surgical procedure, and radiation therapy are significantly associated with survival.

### 3.3 Treatment Factor Analysis

Multivariate Cox regression analysis found that treatment with radiation therapy was associated with an increased 5y OS (HR = 0.613; *p* < 0.001) (Table 3). Univariate analysis showed a similar trend, with patients who received radiation exhibiting improved 5y OS (43.7% [40.84–46.60]) compared to those who did not receive radiation (25.0% [19.43–30.57], *p* < 0.001) (Table 4).

**FIGURE 2:**
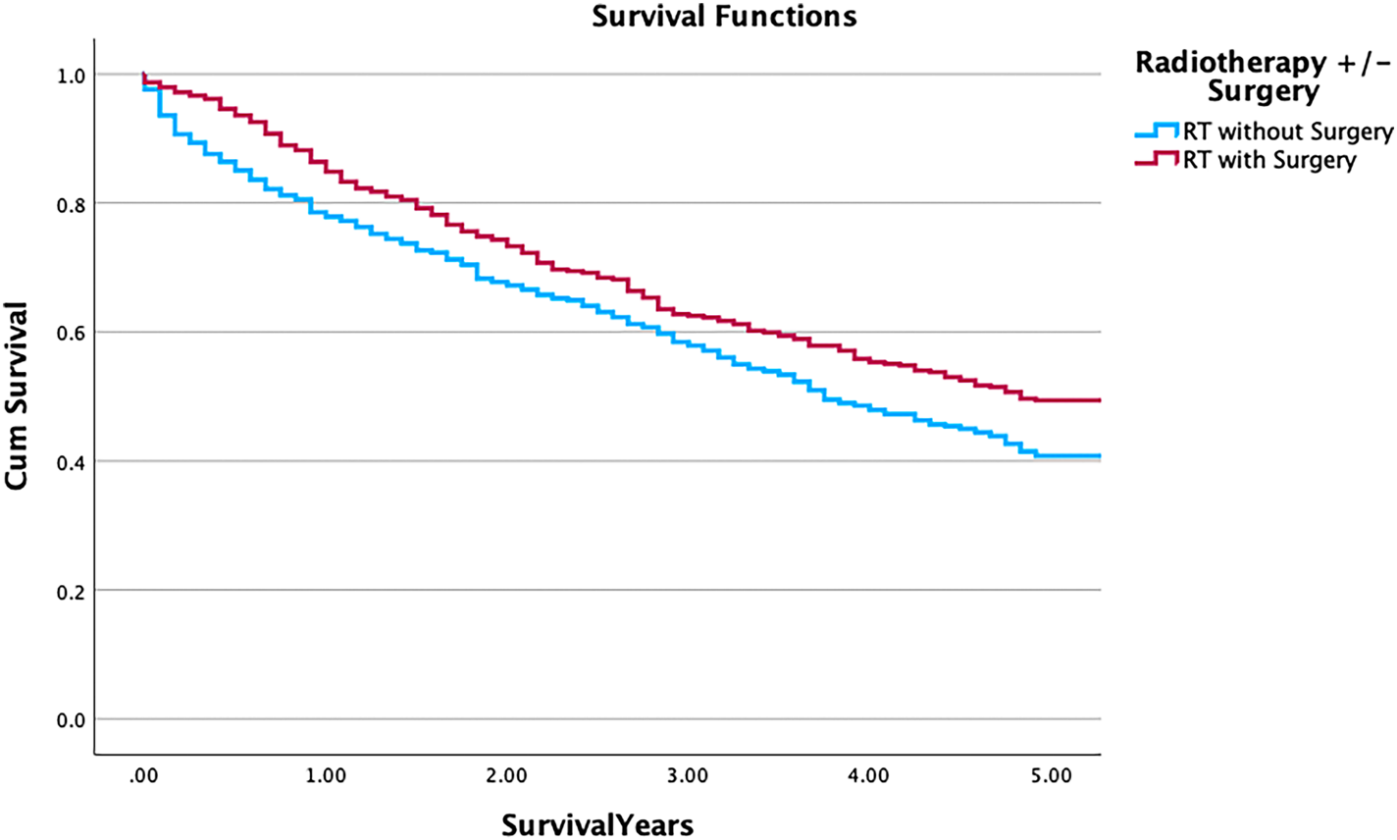
Kaplan–Meier survival OS curve of solitary plasmacytoma of the spine treated with radiation with or without surgery. Kaplan–Meier 5y OS curve illustrating survival outcomes for patients with solitary plasmacytoma of bone of the spine, stratified by treatment modality: radiotherapy with surgery versus radiotherapy without surgery. A significant difference in survival is observed across time points (*p* = 0.004).

Further univariate analysis showed that patients treated with radiation and surgery had increased 5y OS (49.4% [44.39–54.33]) when compared to patients treated with radiation but not surgery (40.8% [37.28–44.32], *p* = 0.004). Figure 2 displays Kaplan–Meier survival curves exemplifying that patients who received RT combined with surgery have improved cumulative survival throughout the five‐year period, with the survival curves separating distinctly from those who received radiation alone (Figure 2). Multivariate Cox regression analysis found no significant association between chemotherapy and overall survival (HR = 0.906; *p* = 0.337) (Table 3). Univariate analysis further demonstrated no statistically significant association between patients treated with chemotherapy (41.7% [35.09–48.24]) and those not treated (40.1% [37.28–42.89]) (*p* = 0.463) (Table 4). Multivariate Cox regression analysis found a complex relationship with surgical procedures and survival but was notably significant as a grouped variable (*p* = 0.013). GTR was associated with improved survival (HR = 0.657; *p* = 0.020). However, surgical procedure NOS was associated with a decreased 5y OS (HR = 1.725; *p* = 0.033). Additionally, tumor destruction and non‐resection were found to be nonsignificant (HR = 0.709; *p* = 0.404) as was subtotal resection (HR = 0.886; *p* = 0.157) (Table 3). A subgroup survival analysis comparing RT‐alone to surgery‐alone revealed higher OS in the surgery group: 59.20% [55.68–62.72] in the RT group and 69.80% [60.06–79.47] in the surgery group, but the log‐rank test did not show a a statistically significant difference (*p* = 0.28) favoring surgery‐alone.

**FIGURE 3:**
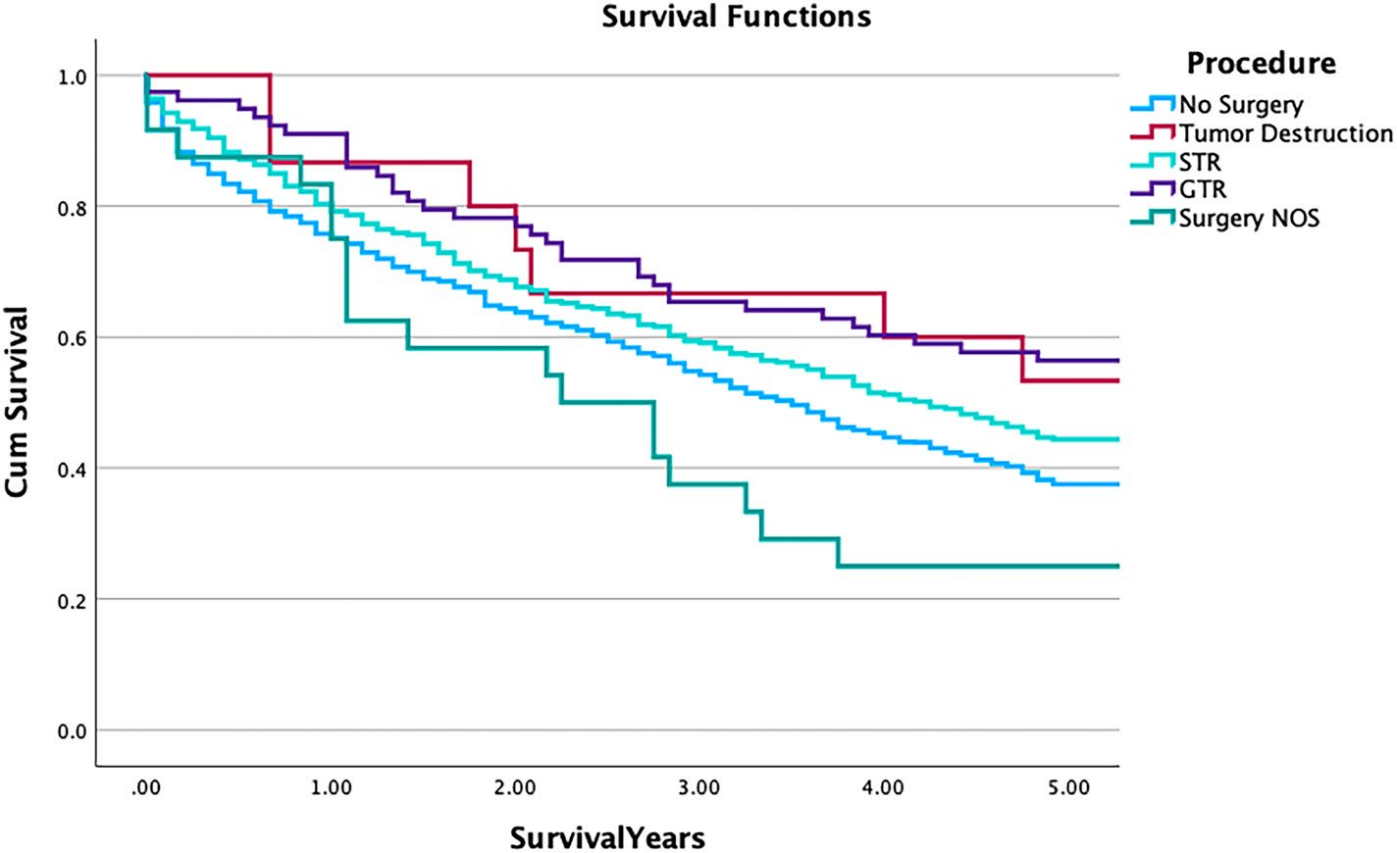
Five‐year overall survival in solitary plasmacytoma of bone of the spine patients stratified by surgical procedure. Kaplan–Meier curves depicting OS for solitary plasmacytoma of bone of the spine patients stratified by surgical procedure. A significant difference in survival is observed across time points (*p* = 0.002).

Univariate analysis demonstrated a trend showing improved 5y OS with surgical intervention, with gross total resection (GTR) having the best survival (56.4% [45.41–67.42]) when compared to no surgery (37.5% [34.37–40.66], *p* = 0.002). The Kaplan–Meier survival curves (Figure 3) illustrate the survival probabilities across five years based on different surgical procedures, with GTR exhibiting the highest cumulative survival and surgery NOS showing the poorest outcomes.

## 4 Discussion

Analysis revealed worse overall survival with increasing age, consistent with findings from related studies on solitary plasmacytoma [5, 7]. A multivariate Cox regression analysis found patients aged 65 years and older at the time of diagnosis demonstrate a HR of 2.25 (*p* < 0.001) compared to younger patients (aged 0–64 years). Survival progressively declines with age in patients with spinal SPB [7]. The findings emphasize the critical need for early awareness, diagnosis, and intervention for SPBS, especially in older patients, who are more vulnerable to poor survival outcomes.

Additionally, the Cox regression analysis demonstrates that the specific type of surgical intervention is highly relevant to OS and should not be overlooked (*p* = 0.013). Specifically, GTR is associated with the highest survival rates, with an HR of 0.657 (*p* = 0.020), corroborating its significance in treatment protocols. This result provides depth and expands on findings made in previous studies, which merely reported superior outcomes for surgical resection compared to non‐surgical resection [2, 5]. Kaplan–Meier analysis further reveals positive outcomes with GTR, with a 5y OS of 56.4% for patients undergoing GTR versus 44.4% for those receiving STR. However, the proportion of SPBS patients undergoing GTR remains low, with only 5.6% receiving this extent of resection. This discrepancy may reflect cases where the tumor was difficult to resect due to its location or other factors, such as differences in tumor biology, patient selection criteria, and access to specialized surgical expertise.

The Cox regression analysis found that RT is associated with a significant improvement in OS, with an HR of 0.613 (*p* < 0.001), particularly when combined with surgery. These results are consistent with previous studies on SPBS, which reported improved outcomes with adjuvant RT [2, 5, 7]. Additional univariate subgroup analysis comparing 5y OS with and without surgery revealed that survival is significantly improved for patients receiving RT combined with surgery compared to RT alone (49.4%–40.8%, *p* = 0.004; Table 4). This significant finding is observed in Figure 2, and it has not been directly studied in other national SEER SPBS analyses, including studies by Chen et al. [5] and Wang et al. [7], which emphasized the prognostic value of early diagnosis and aggressive intervention. The significant improvement in survival from RT and surgery adds clarity for the clinical guidelines established by national treatment of SPBS; the NCCN protocol recommends RT with or without surgery for localized SPBS; however, our significant findings indicate that RT followed by surgery is superior to the alternative, suggesting a potential change to the current national guidelines [6]. Furthermore, this analysis also examined whether surgery‐alone conferred a survival advantage over radiotherapy‐alone. While some recent studies suggest that surgical interventions, either alone or with adjuvant radiotherapy, may offer improved survival outcomes compared to radiotherapy alone, our findings explored this idea but could not contribute significance to this growing body of evidence indicating that RT alone is associated with significantly better survival than surgery alone [11]. It can, however, corroborate that the use of radiotherapy in solitary plasmacytoma is common and has shown high local control rates, with multiple studies reporting response rates of up to 80% [12, 13].

Marital status emerged as a significant predictor of 5y OS in the Cox regression analysis, with married patients exhibiting a survival advantage (HR = 0.671, *p* < 0.001; Table 3). A Kaplan–Meier analysis further reveals that specific marital status subgroups have notable implications for survival (*p* < 0.001). Divorced and widowed patients have lower 5y OS (29.6% and 22.9%, respectively) compared to married, single, never married, and even separated patients (Table 4). This observation that divorced and widowed patients have lower five‐year overall survival, a finding not reported in other studies, provides valuable new insights and contributes to the broader understanding of prognostic factors in this patient population. Generally, these marital status trends align with previous studies, where married individuals are found to have better OS on univariate analysis but without confirmation in a multivariate independent fashion [7]. Additionally, the Cox regression analysis highlights income disparities, with higher‐income patients unexpectedly exhibiting poorer OS (HR = 1.471; *p* < 0.001). These findings suggest that factors beyond economic resources, such as treatment choices and disease awareness, may influence survival in SPBS patients.

This study's utilization of data from the SEER database introduces limitations, including the absence of detailed treatment information, such as RT dosing, chemotherapy regimens, and data on recurrence rates and functional outcomes. Additionally, there could be confounding variables not reported in the SEER database that introduce bias and distort measures of association between exposures and health outcomes. Another limitation is the absence of data on patient comorbidities and performance status, which are important factors influencing treatment selection and survival outcomes but are not captured in the SEER. Furthermore, the findings may not be fully applicable to non‐SEER populations due to potential selection biases. Future research should prioritize prospective studies that incorporate molecular profiling to better stratify patients and guide treatment decisions. Additionally, the development of tailored therapeutic strategies and the exploration of novel biomarkers could significantly enhance outcomes for SPBS patients.

## 5 Conclusion

This study emphasizes the importance of early detection and aggressive treatment with radiotherapy combined with surgery, specifically in conjunction with GTR of the tumor, for improved survival among patients with plasmacytoma of the spine. Our findings, particularly the superiority of radiotherapy combined with surgery, suggest potential changes to the NCCN guidelines. To the authors' knowledge, this is the first paper to report on the extent of surgery improving survival among patients with plasmacytoma of the spine. Additionally, we identify novel prognostic factors, such as the impact of marital status on survival, particularly with worsened 5y OS in widowed and divorced patients. Furthermore, income disparities highlight that factors beyond economic resources may affect survival. Due to limitations in SEER data, including a lack of detailed individualized treatment and recurrence information, future studies should focus on molecular profiling and tailored treatments to improve outcomes. Continued research into biomarkers and therapeutic strategies is warranted to advance SPBS care and survival.

## Author Contributions

All authors contributed to the study conception and design. Material preparation, data collection, and analysis were performed by Kevin E. Agner and Luke G. Comisford. The first draft of the manuscript was written by Alec G. Kotler, Jacob A. Wells, Michael C. Larkins, and Kevin E. Agner, and all authors commented on previous versions of the manuscript. All authors read and approved the final manuscript.

## Ethics Statement

The authors have nothing to report.

## Consent

The authors have nothing to report.

## Conflicts of Interest

The authors declare no conflicts of interest.

## Data Availability

The data that support the findings of this study are openly available in Surveillance, Epidemiology, and End Results (SEER) Program at https://seer.cancer.gov/.
